# Facile Preparation of Chitosan-Based Composite Film with Good Mechanical Strength and Flame Retardancy

**DOI:** 10.3390/polym14071337

**Published:** 2022-03-25

**Authors:** Jirui Chen, Wentao Huang, Yifan Chen, Zenan Zhou, Huan Liu, Wenbiao Zhang, Jingda Huang

**Affiliations:** 1 College of Chemistry and Materials Engineering, Zhejiang A&F University, Hangzhou 311300, China; chenjirui1991@163.com (J.C.); h0106w@163.com (W.H.); d_chen666@126.com (Y.C.); zhouzenan@163.com (Z.Z.); cooked123@126.com (H.L.); 2 China Bamboo Charcoal Museum, Lishui 323300, China

**Keywords:** chitosan, cellulose nanofiber, flame retardant, film, mechanical strength

## Abstract

To improve on the poor strength and flame retardancy of a chitosan (CS)-based functional film, cellulose nanofiber (CNF) was taken as the reinforced material and both ammonium polyphosphate (APP) and branched polyethyleneimine (BPEI) as the flame-retardant additives in the CS matrix to prepare the CS/CNF/APP/BPEI composite film by simple drying. The resulting composite film showed good mechanical strength, with a tensile strength reaching 71.84 Mpa due to the high flexibility of CNF and the combination of CS, CNF and BPEI through strong hydrogen bonding interactions. The flame retardant-performance of the composite film greatly enhanced the limit oxygen index (LOI), up to 32.7% from 27.6% for the pure film, and the PHRR intensity decreased to 28.87 W/g from 39.38% in the micro-scale combustion calorimetry (MCC) test due to the ability of BPEI to stimulate the decomposition of APP, releasing non-flammable gases such as CO_2_, N_2_, NH_3_, etc., and forming a protective phosphating layer to block the entry of O_2_. Based on the good flame retardancy, mechanical strength and transparency, the CS/CNF/APP/BPEI composite film has a great potential for future applications.

## 1. Introduction

Thin film materials are used in all areas of life. However, they are mainly made of synthetic polymers such as polyethylene [1], polypropylene [2], polyvinylchloride [3], polycarbonate [4], etc., These synthetic polymers are difficult to degrade and would lead to a burden on the environment. Therefore, there is much interest in the development of functional films using natural degradable polymers.

Chitosan (CS), as a deacetylated chitin, is an abundant and renewable natural polymer. CS has excellent film-forming properties, environmental sustainability, and selective permeability to oxygen and carbon dioxide in the air, showing its good prospects in the thin film field [5,6,7]. For example, after a CS film is attached onto the surface of carton, the storage life of mango can be improved [8]. CS is often compounded with other inorganic materials to prepare composite CS-based functional films, such as CS-SiO_2_ [9], CS-TiO_2_ [10], etc. [11]. However, the introduction of these inorganic particles blocks the formation of hydrogen bonds among CS molecules around them and reduce their mechanical properties. Consequently, there is a lot of research focusing on improving their mechanical strength. For example, the mechanical strength of the CS film can be enhanced by the addition of tetraethoxysilane/vinyltriethoxysilane [12]. Carbon nanotubes are often used to improve the mechanical properties of the CS-based films, but the transparency becomes poor [13]. The reinforcement materials referred to above have poor environmental performance, and even affect the film’s transparency. Cellulose nanofiber (CNF), a renewable green material, is mainly stripped from wood, cotton, straw, etc., and widely used for the enhancement of film materials due to its high strength, good biocompatibility and environmentally friendly performance [14,15,16]. For example, CNF was added to a hemicellulose film rich in xylan and greatly improved the mechanical properties [17]. Additionally, a poly (vinyl alcohol) (PVA) film with the addition of CNF also showed good mechanical strength [18].

Fire hazard prevention has always been a focus of social attention and the research and development of flame-retardant materials are sought after by scholars, with more and more attention being paid to the flame-retardant performance of biomass films [19]. CS itself has a certain flame-retardant performance due to the presence of both C and N elements, but it is difficult for CS to achieve a high-efficiency and stable flame-retardant effect without combination with other flame-retardant materials [20]. In addition, when CS was composited with a combustible material in our preliminary burning test, flame was observed to break out during the burning process, bringing a certain fire risk in practical application. To enhance the flame-retardant grade and stability of the CS-based films, some flame retardants, such as chlorine flame retardants, bromine flame retardants, phosphorus flame retardants, inorganic flame retardants, etc., were added to the CS matrix [21]. Among them, the organophosphorus flame retardants have a good development prospect due to their advantages of low smoke production, non-toxicity, and low halogen content [22]. In particular, APP and BPEI are a common combination in organophosphorus flame retardants. Through the thermal decomposition process, APP as the acid can generate stable polyphosphoric acid and cut off the supply of oxygen; BPEI as a foaming agent and carbon source can release nonflammable gas and produce a loose carbon protective layer. For example, a flame retardant cotton fabric was prepared by spraying the coating solution containing APP and BPEI onto the cotton fabric [23]. Analogously, BPEI, APP and fluorodecyl polyhedral polysiloxane were deposited on the surface of the cotton fabric by simple layer-by-layer self-assembly; the resulting cotton showed excellent flame retardant properties, superhydrophobicity and self-healing properties [24]. Our research group also achieved a good flame retardant effect on the cotton fabrics through the introduction of both APP and BPEI [25].

The mechanical strength of CS-based films is often reduced by the addition of some functional particles and the flame retardant performance also still need to be improved. In this study, in order to produce a CS-based film with good mechanical strength and flame retardancy, CNF was used as the reinforcement material to improve the mechanical properties of CS films; both APP and BPEI were used to enhance the flame retardant performance of CS-based films. The resulting CS/CNF/APP/BPEI composite film was prepared by simple drying. In view of the excellent properties and simple preparation process, the resulting composite film has good prospects for potential application.

## 2. Materials and Methods

### 2.1. Materials

Chitosan (CS, Deacetylation degree of ~95%, viscosity of 100–200 MPa.S) and Glacial acetic acid (AR, 99.5%) were purchased from Macklin Biochemical Technology Co., LTD, Shanghai, China. Cellulose nanofiber (CNF, solid content of 1.03%, purity of ~99%) was purchased from Tianjin Woodelf Biotechnology Co. Ltd., Tianjin, China. Both branched poly (ethylenimine) (BPEI, average Mw of ~800 by LS, average Mn of ~600 by GPC) and ammonium polyphosphate (APP, *n* > 1000) were purchased from Sigma (Shanghai, China).

### 2.2. Preparation of Flame Retardant CS-Based Composite Film

First, CS of 1.5 g was added to glacial acetic acid solution (1 wt%) of 100 mL in a beaker, followed by stirring until the CS completely dissolved (as described in the report [26,27]). The CNF aqueous suspension (modulated to 0.8 wt%) of 0.2 g was added to the above CS solution and dispersed by ultrasonic treatment at 500 w for 5 min to obtain the CS/CNF aqueous suspension. Subsequently, BPEI of 0.1 g was put into a beaker with deionized water of 50 mL and stirred until completely dissolved. Then, APP powder of 0.1 g was added to the BPEI solution and stirred until the APP powder was uniformly dispersed to obtain the APP/BPEI aqueous suspension, followed by dropping 5 mL of APP/BEPI mixture into CS/CNF solution. Next, both CS/CNF and APP/BPEI aqueous suspensions were mixed by stirring at 800 rap/min for 1 h to form a uniform CS/CNF/BPEI/APP mixture. Finally, a certain amount of the CS/CNF/APP/BPEI mixture was dropped into a Petri dish with a diameter of 6 cm, and then dried in a vacuum drying oven of 30 °C until the water was completely evaporated to form a CS/CNF/APP/ BPEI composite film. The thickness of the CS-based composite film was 0.08 ± 0.01 mm and was roughly determined by controlling the amount of the mixture in the Petri dish. For comparison, the pure CS film and CS/CNF composite film without APP/BPEI were made by the same method.

### 2.3. Mechanical Properties Test

According to previous reports [28], the samples were cut into the sizes of 3 mm × 1 mm × 0.08 ± 0.01 mm (length × width × height) for the tensile strength test with a span of 100 mm and a compression rate of 3 mm/min by a universal mechanical testing machine (598X, Instron company, Norwood, MA, USA).

### 2.4. LOI Test

The LOI value of the CS-based samples with a size of 150 mm × 10 mm × 0.08 mm ± 0.01 mm (length × width × height) was estimated using an oxygen index meter (JF-3, Jiangning District Analytical Instrument Factory, Nanjing, China), where both oxygen and nitrogen were used to control the air atmosphere by adjusting the flow of each. The result LOI value is an average value of three samples tested.

### 2.5. MCC Test

A micro-scale combustion calorimetry (MODEL-MCC-2, Govmark, Farmingdale, NY, USA) was used to evaluate the combustion performance of the CS-based samples. The usage amount of each sample was 5 mg, the temperature range was set from 30 °C to 750 °C. At the same time, the heating rate was 5 °C/min.

### 2.6. Characterization

The microstructure of the CS-based composite films was observed by scanning electron microscopy (SEM, Hitachi S-4800). A Fourier transform infrared spectroscopy (FTIR, Perkin Elmer, USA) was used to analyze the chemical compositions of the different CS-based composite films, with a range of 400–4000 cm^−1^ across 32 scans. The crystal structure of the different CS-based composite films was detected by X-ray diffraction (XRD-6000, Shimadzu company, Tokyo, Japan) with 2θ from 5° to 65°, and the crystallinity of the CS-based composite films was analyzed using MDI JADE XRD spectrum analysis software by separating and fitting the peaks of the XRD spectrum. The thermostability of the different CS-based composite films was analyzed using thermogravimetric analysis with a temperature range of 25~800 ℃ (TG- STA 449F3, Netzsch, Germany).

## 3. Results and Discussion

### 3.1. Formation Mechanism of CS-Based Composite Film

**Figure 1:**
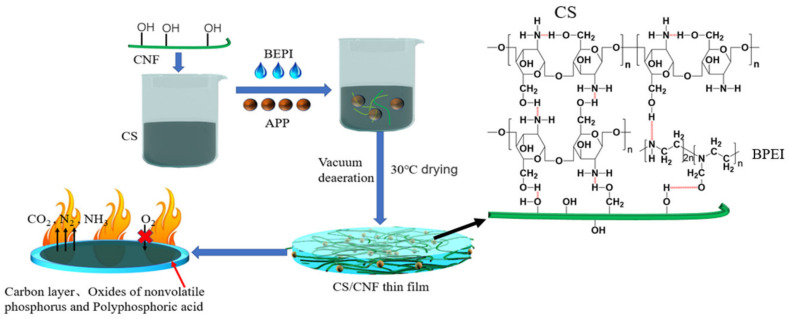
Formation mechanism of CS-based composite film.

As shown in Figure 1, the preparation of the CS-based composite films was simple and facile. CNF, BPEI and APP were added to the dissolved CS matrix; then, the composite film was formed by drying. CS, a processed product of natural polysaccharide chitin after further removing acetyl group, is non-toxic, pollution-free, and biodegradable, and has high surface activity due to the rich -OH and -NH_2_ groups on its molecular chain. As the solvent evaporates, these active groups (-OH and -NH_2_) gradually become closer. They can form intramolecular hydrogen bonds, as well as intermolecular hydrogen bonds with that of the adjacent CS molecular chain (as shown in the chemical structural formula of Figure 1). These hydrogen bonds make CS molecules easily form crystalline phase regions, resulting in a good film-forming property. CNF has a similar structure to CS and possesses a good mechanical strength and high length–diameter ratio. Therefore, CNFs can be entangled with each other to form a stable three-dimensional network in the CS matrix, beneficial to the improvement of mechanical strength. In addition, there are amounts of -OH on the surface of CNF and they can form strong hydrogen bonds with the -OH and -NH_2_ of CS molecular chain and -NH_2_ of BPEI around them (as shown in the chemical structural formula of Figure 1), further enhancing the mechanical strength of the CS/CNF composite film. CS itself contains a large amount of C element and low content of *n* element (about 8.7% according to the report [29]) and has a certain flame retardant effect. It is often used in combination with other flame retardants, and plays an auxiliary flame retardant role. Both APP and BPEI were added to CS/CNF mixture to enhance its flame retardant properties [30,31]. Since both CS and BPEI are cationic polymers and APP is negatively charged, they can attract each other and bind together. In the presence of fire, APP as the acid source generates a stable polyphosphoric acid, which plays a role of oxygen isolation [32]. BPEI as the blowing agent stimulates the decomposition of APP and, as the carbon source, produces a loose carbon layer and typical nonflammable gases (e.g., CO_2_, N_2_, NH_3_). These gases can further block the supply of O_2_, achieving a flame retardant effect. In the composite system, both CS and BPEI are highly viscous and can enhance the bond between CNF and APP. Consequently, a CS-based composite film with good flame retardancy and mechanical strength can be prepared.

### 3.2. Surface Morphologies

**Figure 2:**
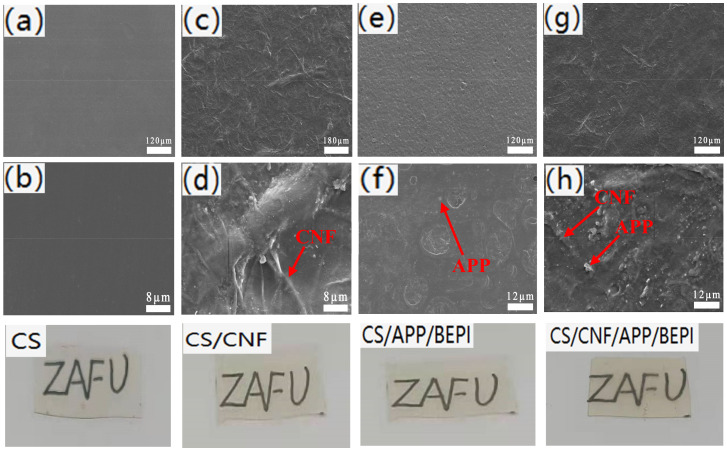
SEM images of the (**a**,**b**) pure CS, (**c**,**d**) CS/CNF, (**e**,**f**) CS/APP/BPEI, and (**g**,**h**) CS/CNF/APP/BPEI composite films and their illustrations showing transparency.

As shown in Figure 2a,b, CS showed good film-forming property, and the pure CS film was smooth and dense due to the strong hydroxyl bonds formed inside and among the CS molecular chains. After the introduction of CNF, the surface of the CS/CNF composite film showed many irregular filamentous bulges due to the partial exposure of CNFs with high length–diameter ratio on the film surface (Figure 2c,d). When only adding both APP and BPEI (Figure 2e,f), there was no obvious phase separation in the composite film, indicating that CS has good compatibility with PEI. However, some small holes appeared on the film surface; this might be caused by bubbles bursting during drying. After the CNF, APP and BPEI were added (Figure 2g,h), the exposures of the CNFs and APP were more obvious. The additional amounts of CNF, APP and BPEI was very small and their distribution was relatively uniform, so the transparency of the CS-based composite films was very good and the covered substrate can be clearly seen in the illustrations of Figure 2.

### 3.3. Chemical Structure Analysis

**Figure 3:**
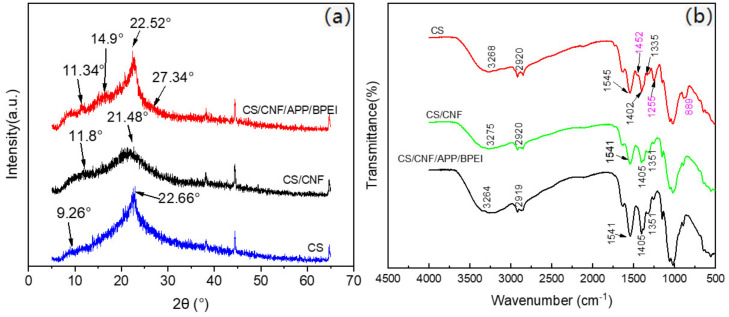
(**a**) XRD and (**b**) FTIR spectrums of the different CS-based composite films.

As shown in Figure 3a, in the pure CS film, the main diffraction peaks are at 9.26° and 22.66°. This is consistent with the previous report [33] where the crystalline forms of CS appear 2θ = about 10° (marked as Form I) and 20° (marked as Form II), respectively. The crystallinity of the CS film as given by the MDI-JADE XRD spectrum analysis software was 22.97%. In the CS/CNF film, the main diffraction peaks were located at 11.8° and 21.48°; the position of the peaks produced a little deviation and the crystallinity of the CS/CNF composite film was reduced to 12.34%. This may be because the hydrogen bonding between CS molecules and CNF disturbs the original crystallization of CS. However, the two peaks of the CS/CNF film widened, which may be due to the superposition of the diffraction peaks of CS and CNF [34]. When both APP and BPEI were added to the CS/CNF system, the characteristic peaks of APP showed at 14.9°and 27.34° [35]. The main diffraction peaks of CS were located at 11.34° and 22.52°, and only had a weaker change compared with that of the CS film, and the crystallinity of the CS/CNF/ APP/BPEI composite film increased to 23.88%. This was because APP could play a role in blocking CS and CNF off from direct contact to some extent and affect the forming of the hydrogen bonds between CS and CNF, weakening the influence of CNF on the crystal structure of the CS film.

FTIR was used to detect the surface chemical structure of the CS-based composite films. As shown in Figure 3b, in the pure CS film the wide absorption peak at about 3275 cm^−1^ corresponded to the stretching vibration of -OH and -NH and the absorption peak at 2900 cm^−1^ was caused by the stretching vibration of C-H; the peak at about 1200 cm^−1^ was formed due to the stretching vibration of C-O; the absorption peaks of 1650 cm^−1^ and 1541 cm^−1^ were produced by the stretching vibration of C=O in the amide group and the deformation vibration of -NH_2_, respectively [36]. After the addition of CNF, the FTIR curve of the CS/CNF composite film was extremely similar to that of the pure film, due to their analogous molecular structure. Compared with that of the pure one, the characteristic peak of both -OH and -NH in the CS/CNF film appeared at 3264 cm^−1^ and moved towards a lower wave number due to the strengthening of intramolecular and intermolecular hydrogen bonds, suggesting that there was a strong hydrogen bond between the CNF and CS. It could be proved that CS and CNF have good biocompatibility, which is beneficial to the stability of the composite film [37]. In the CS/CNF/APP/BPEI composite film, aside from the two peaks at 1452 cm^−1^ and 889 cm^−1^, which were caused by the stretching vibration of -CH_2_ in BPEI and the stretching vibration of P-O-P in APP [38], respectively, there were no new peaks appearing, indicating that there was no chemical reaction between BPEI/APP and CS/CNF, but only physical bonding. This was also consistent with the above formation mechanism analysis results.

### 3.4. Thermal Stability Analysis

**Figure 4:**
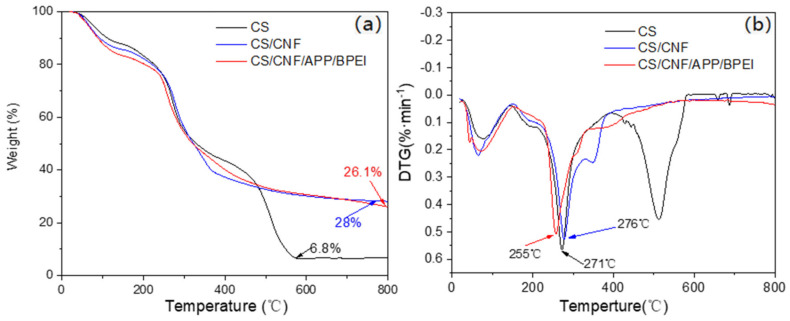
(**a**) TG and (**b**) DTG curve of the different CS-based composite films.

The thermal stability is one of the important indicators of fire retardant properties. Figure 4a showed a change in the CS-based composite films from 25 to 800 °C. The decrease in the mass of all the CS-based composite films below 100 °C was caused by a large amount of evaporation of water. The decomposition and carbonization of CS at around 171~571 °C resulted in a rapid decrease in the mass of the pure CS film, and it exhibited stability after 571 °C because the remaining carbon would not significantly reduce with the change in temperature [39] and the residue was only 6.8% (Table 1). The mass of the CS/CNF composite film decreased from 172 to 377 °C due to the decomposition and carbonization of both CS and CNF. When the temperature reached 400 °C, the CS/CNF composite film tended to be stable in mass. This might be because after the thermal decomposition, the CS formed a carbon layer covering the surface of CNF, which had a certain protective effect. Consequently, the remaining weight of the CS/CNF film was higher [40]. The fastest decomposition rate of the CS-based composite films showed no discernible difference before and after the addition of CNF due to the similar properties of CS and CNF, occurring at 271 °C and 276 °C, respectively (Figure 4b). In the CS/CNF/APP/BPEI composite film, CS, APP and BPEI decomposed with the increase in temperature and produced a loose carbon layer on the CNF surface. Therefore, the TG curve was similar to the CS/CNF one, but showed the lowest temperature (255 °C) of the fastest decomposition rate due to the thermal decomposition of APP and BPEI. After 600 °C, the CS/CNF and CS/CNF/APP/BPEI composite films had higher residual mass (28% and 26.1%, respectively) than that of the pure one, suggesting the addition of CNF, APP and BPEI was beneficial to the thermal stability of the CS-based composite film [25].

### 3.5. Flame Retardant Analysis Test

**Figure 5:**
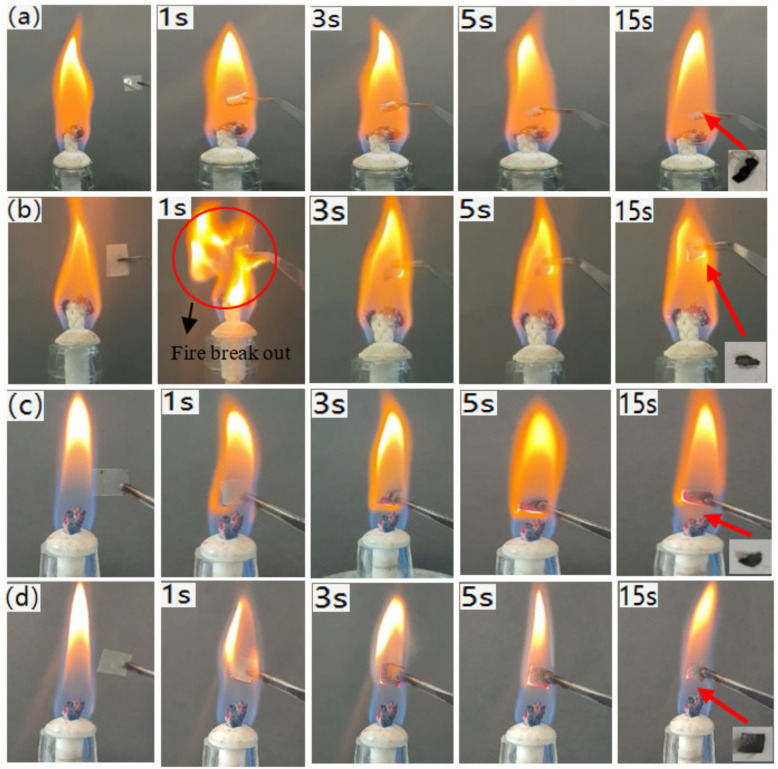
Flame burning test of the (**a**) CS film; (**b**) CS/CNF film; (**c**) CS/APP/BEPI film; (**d**) CS/CNF/APP/BEPI film.

In the flame burning test, the samples, with a weight of about 10 mg, were moved into a flame from an alcohol lamp and not moved out until 15 s of burning was observed [41]. Figure 5 shows the macroscopic morphologies of the CS-based composite films during the process of the combustion test. The pure CS film curled up when entering the flame from the alcohol lamp and there was a small amount of burning residue left after 15 s (Figure 5a). After the addition of CNF, flame was observed to break out (Figure 5b), which might cause certain harm in the practical application, but not in the CS/APP/BPEI composite film (Figure 5c). This was caused by the combustion of CNF on the composite film surface. When CNF, APP and BPEI were added, no flame was observed (Figure 5d). This was because the CNF was covered by BPEI and APP and became unburnable.

**Figure 6:**
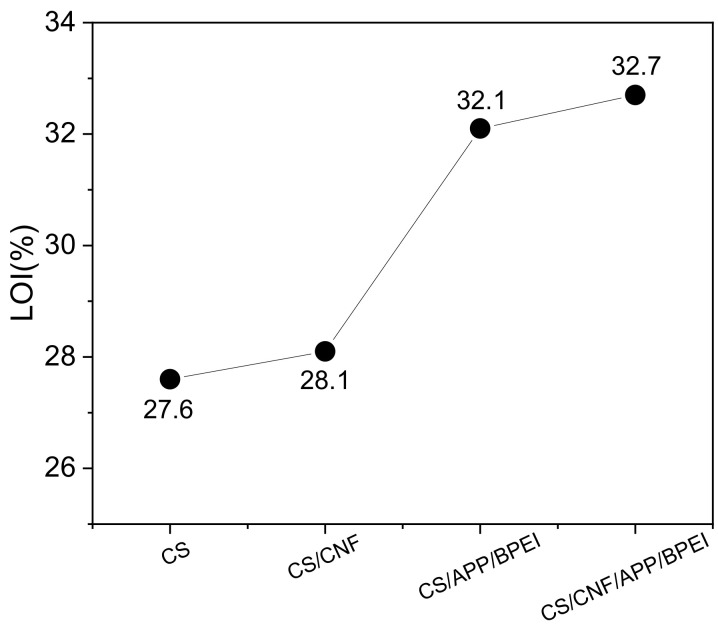
LOI curve of the different CS-based composite films.

The LOI value is one of the key indicators of flame-retardant materials; when its LOI value is not less than 21%, it is called a flame-retardant material [42]. The samples were ignited in an LOI meter, the ignition source was immediately removed, and their burning was observed. The results (Figure 6) showed that the LOI value of the pure CS film was 27.6%. CS itself had a certain flame retardant property due to its high carbon content and a portion of *n*, as well as some nonflammable gases and carbon layers that were formed during heating, which helped prevent further combustion. The LOI value of the CS/CNF film was 28.1% and showed no significant difference compared to the pure CS film, which might be due to the low additional amount of CNF. After the addition of both APP and BPEI, the flame retardant performance of the CS/APP/BPEI and CS/CNF/APP/BPEI composite films had been greatly improved and their LOI values were up to 32.1% and 32.7%, respectively. This was because APP and BPEI increased the *N* and *P* elements of the system, and BPEI could stimulate the decomposition of APP, releasing CO_2_, N_2_, NH_3_, etc., and forming a protective phosphating layer to block the entry of O_2_.

**Figure 7:**
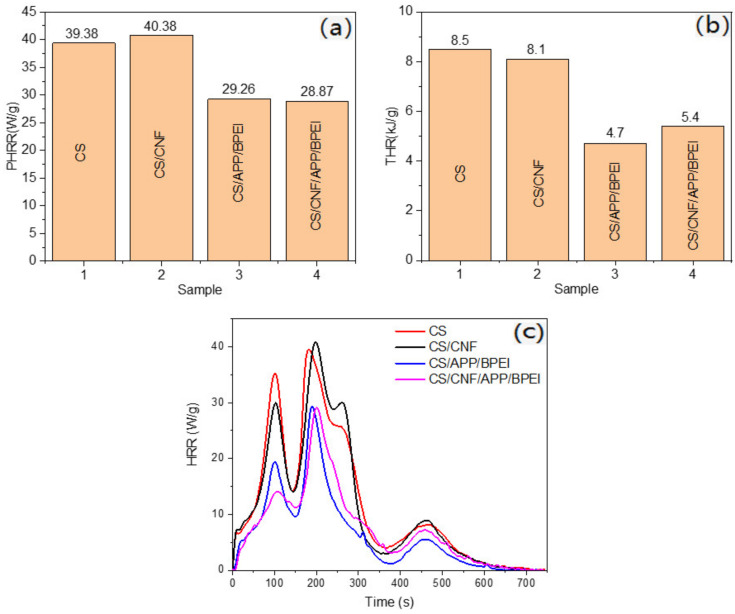
The (**a**) PHHR capacity comparison, (**b**) TRR comparison, (**c**) HRR curves of the different CS-based composite films.

To further verify the flame retardant properties, the MCC analysis was conducted. As shown in Figure 7a–c, it could be seen that both before and after adding only CNF, the heat release rate (HRR), the peak heat release rate (PHRR) and the total heat release rate (THR) all showed no discernible difference due to the low additional amount of CNF. Additionally, the CS-based films showed the strong peaks at about 210 °C, and the corresponding PHRR reached 39.38 W/g and 40.83 W/g before and after adding only CNF, indicating that there was no obvious effect on flame retardancy. After adding APP/BPEI, the HRR, PHRR and THR showed a significant decrease, and the peak intensity (PHRR) of the CS/APP/BPEI and CS/CNF/APP/BPEI composite films decreased significantly to 29.26 W/g and 28.87 W/g, respectively, proving that the flame retardant effect had greatly improved. The results corresponded with the LOI value.

### 3.6. Mechanical Properties Test

**Figure 8:**
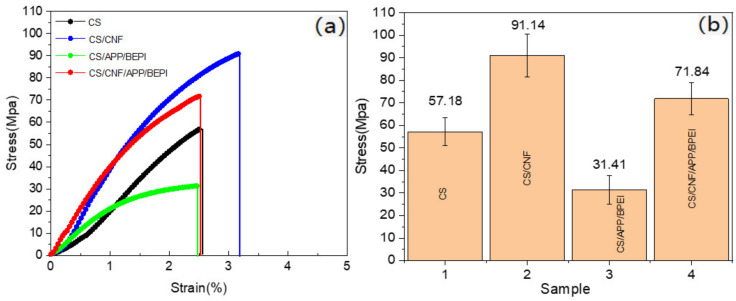
The (**a**) stress–strain curves and (**b**) tensile strength of the different CS-based composite films.

As shown in Figure 8a,b, the pure CS film had good ductility and its tensile strength was 57.18 Mpa because of CS’s long chain molecular structure. They displayed very good film-forming due to the interaction of hydrogen bonding (as described above Figure 1). When APP and BPEI were increased, its ductility became poor and the tensile strength was down to 31.41 Mpa, as APP, as a granular material, was able to block the hydrogen bond links among CS molecular chains [43]. There were no chemical bonds with CS and PEI (as analyzed in Figure 4), which affected the stability of the whole film. Therefore, CNF was used to inhibit the effect of APP on the mechanical properties of CS film. The mechanical strength of the CS/CNF composite film showed a great improvement and reached 91.14 Mpa. This is because CNF is a filamentous material and easily able to form a stable three-dimensional network structure in the CS matrix [38]. Additionally, the abundant -OH on the CNF surface could form strong hydrogen bonds with the active groups of CS. However, the ductility became weak. When CNF was added to the CS/APP/BPEI system, the tensile strength came back to 71.84 Mpa, showing a good inhibitory effect on the negative impact of APP on the mechanical strength of CS-based films.

## 4. Conclusions

In summary, a CS-based composite film with good flame retardancy, mechanical strength and transparency could be prepared by a simple process of mixing CS, CNF, APP and BPEI, and drying. It was found that CNF is an ideal natural material for improving the mechanical properties of the CS-based film; the mechanical strength of the CS/CNF composite film reached 91.14 Mpa and was much higher than the pure CS film (57.18 Mpa). This is because CNF itself has a high toughness and is rich in surface hydroxyl groups which can produce strong hydrogen bonding with CS. Both APP and BPEI are highly effective and commonly used flame retardants and could greatly enhance the flame retardant effect and the thermal stability of the CS-based composite film, in which the LOI value was up to 32.7% compared to 27.6% in the pure CS film. To a certain extent, APP particles could inhibit the formation of hydrogen bond among CS, CNF and BPEI, and cause a decrease in the mechanical strength, but it was still at 71.84 Mpa due to the present of CNF. The facile preparation strategy and good performance means the CS-based composite film has good prospects for development.

## Figures and Tables

**Table 1:** Decomposition temperature and residue of the CS-based films.

Sample	Decomposition Temperature (°C)	Residue at 800 °C (wt%)
CS	205.41	6.8
CS/CNF	210.27	28
CS/CNF/APP/BPEI	220.27	26.1

## Data Availability

The data presented in this study is available on request from the corresponding author.
